# Vertical Ridge Augmentation of the Atrophic Posterior Mandible with Sandwich Technique: Bone Block from the Chin Area versus Corticocancellous Bone Block Allograft—Clinical and Histological Prospective Randomized Controlled Study

**DOI:** 10.1155/2014/982104

**Published:** 2014-04-29

**Authors:** Luigi Laino, Giovanna Iezzi, Adriano Piattelli, Lorenzo Lo Muzio, Marco Cicciù

**Affiliations:** ^1^Department of Clinical and Experimental Medicine, University of Foggia, FO, Italy; ^2^Department of Stomatology and Oral Science, University of Chieti, Italy; ^3^Human Pathology Department, School of Dentistry, University of Messina, Via Consolare Valeria, 98100 Messina, Italy

## Abstract

The aim of the present study is to compare the histological aspects of bone formation in atrophic posterior mandibles augmented by autologous bone block from chin area with corticocancellous bone block allograft used as inlays with the sandwich technique. * Materials and Methods*. Sixteen patients with bilateral partial edentulism in the posterior mandible were selected. The residual bone height, preliminarily measured by computed tomography scans, ranged between 5 and 7 mm from the inferior alveolar nerve. All patients required regeneration procedure with autologous bone block from chin area (control group) versus bone block allograft Puros (Zimmer Dental, 1900 Aston Avenue, Carlsbad, CA, USA) (test group). Histological and histomorphometric samples were collected at the time of implant positioning in order to analyze the percentage of newly formed bone, the residual graft material, and marrow spaces/soft tissue. * Results*. No statistically significant differences between the two groups were found regarding the percentage of newly formed bone. The percentage of residual grafted material was significantly higher in the test group, whilst the percentage of marrow spaces was higher in control group. * Conclusions*. In conclusion, both procedures supported good results, although the use of bone blocks allograft was less invasive and preferable than harvesting bone from the mental symphysis.

## 1. Introduction

The rehabilitation of posterior mandible with dental implants represents today a hard challenge for clinicians due to the lack of supporting bone. The alveolar nerve presence and lifting and the gradual vertical and horizontal resorption of the mandibular bone crest in both partially and totally edentulous patients can be treated by several prosthetic and surgical options [1–5]. Patients can be rehabilitated with conventional partial removable dentures, but often this treatment does not meet the expectations of the patients. Regarding implant supported treatment options, vertical ridge augmentation, surgical displacement of the inferior alveolar nerve, and, finally, the placement of short implants (8 mm or less) could be necessary for the correction of the atrophic posterior mandible. The use of short implants represents a simpler and faster alternative to the augmentation procedure, even if in some “critical cases” the residual bone crest above the inferior alveolar nerve is only 5–7 mm in height, and therefore the surgical augmentation treatment is mandatory. Indeed, the displacement of the alveolar nerve is technically tough, and this procedure may be associated with certain degree of permanent loss of nerve sensitivity [1, 6–9]. Different surgical techniques are currently being used to augment the posterior mandible: guided bone regeneration (GBR) and alveolar distraction osteogenesis onlay bone grafting; however, only few of these have been tested in randomized clinical trial (RCT) [10, 11]. Several surgical bone augmentation techniques are related to an unpredictable resorption of the grafted material. Vascularity seems to be the main factor in determining whether such a graft can be maintained in situ. Traditional distraction osteogenesis aims to maintain the majority of the vascularity to the transported bone segment. The drawbacks of distraction osteogenesis include patient cooperation, technique sensitivity, and the possibility of a second surgery to remove the device [1, 3, 7].

Another possible approach is to use an interpositional bone graft [1, 4, 8, 11]. The rationale of the interpositional techniques is based on the theory that biomaterial placed between 2 pieces of pedicled bone with internal cancellous bone will undergo rapid and complete healing and graft incorporation with a lower percentage of resorption. The sandwich osteotomy allows for the positioning of the graft in a well-delimited area as well as offering adequate blood supply to maintain new bone growth. This procedure enables the simultaneous correction of the sagittal intermaxillary relationship and the vertical dimension. This technique has been used in a variety of maxillary areas including both the anterior and posterior mandible and maxilla. When performing the sandwich osteotomy in the posterior mandible, great surgical precision is required to avoid damage to the inferior alveolar nerve. For these reasons and for the few results available in the literature, it is necessary to carry out further research to validate the predictability of this regenerative technique [1, 3, 8–11].

The aim of the present study is to compare the histological aspects of bone formation in atrophic posterior mandibles augmented by autologous bone block from chin area (control group) to Puros bone block allograft (test group) used as inlays with the sandwich technique.

## 2. Materials and Methods

Between November and April 2010, nineteen patients with bilateral partial edentulism in the posterior mandible were selected for the present study. They all showed a residual bone height ranging between 5 and 7 mm from the inferior alveolar nerve, which was firstly measured by computed tomography scans. All patients required the placement of at least 3 implants. The protocol of the study was approved by the Ethical Committee of the Second University of Naples, Naples, Italy, and all the patients signed a written informed consent form. All patients were treated in the Department of Oral and Maxillofacial Surgery, Second University of Naples, Naples, Italy. Exclusion criteria were (1) general contraindications to implant surgery, (2) irradiation, chemotherapy, or immunosuppressive therapy over the past 5 years, (3) poor oral hygiene and motivation, (4) active periodontitis, (5) uncontrolled diabetes, (6) pregnancy or lactation, (7) substance abusers, (8) smoking more than 10 cigarettes per day, (9) psychiatric problems or unrealistic expectations, (10) acute infection in the area intended for implant placement, (11) positive to HIV and hepatitis B and C, (12) autoimmune diseases such as rheumatoid arthritis, systemic lupus erythematosus, scleroderma, Sjogren's syndrome, and dermatomyositis/polymyositis, (13) treated or under treatment with intravenous aminobisphosphonates, (14) previously subjected to reconstructive procedures of the posterior mandible, and (15) under chronic treatment with steroids or nonsteroidal anti-inflammatory drugs. Twelve patients were considered eligible and were enrolled in the trial (mean age was 57 years, 9 females and 3 males).

### 2.1. Augmentation Procedure

**Figure 1:**
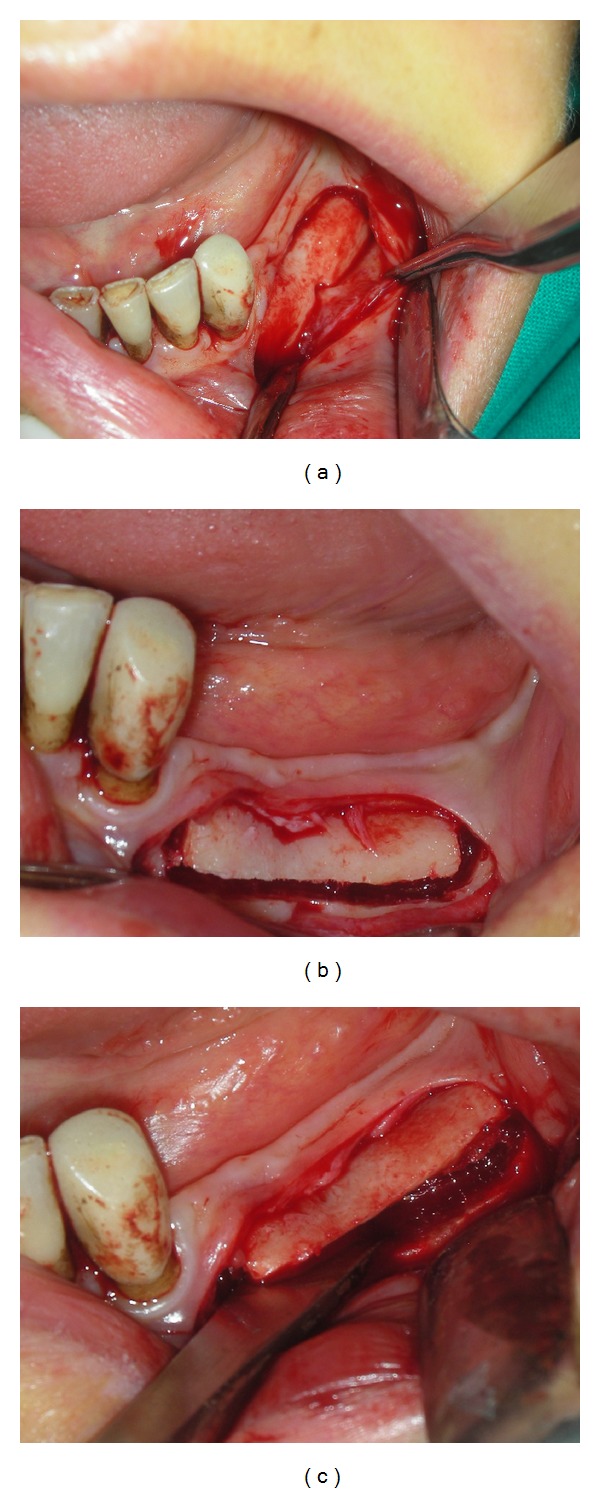
Sample of the two oblique cuts performed in the coronal third portion of the mandibular bone with the mesial cut at least 2 mm distal to the last tooth in the arch (a, b, c).

**Figure 2:**
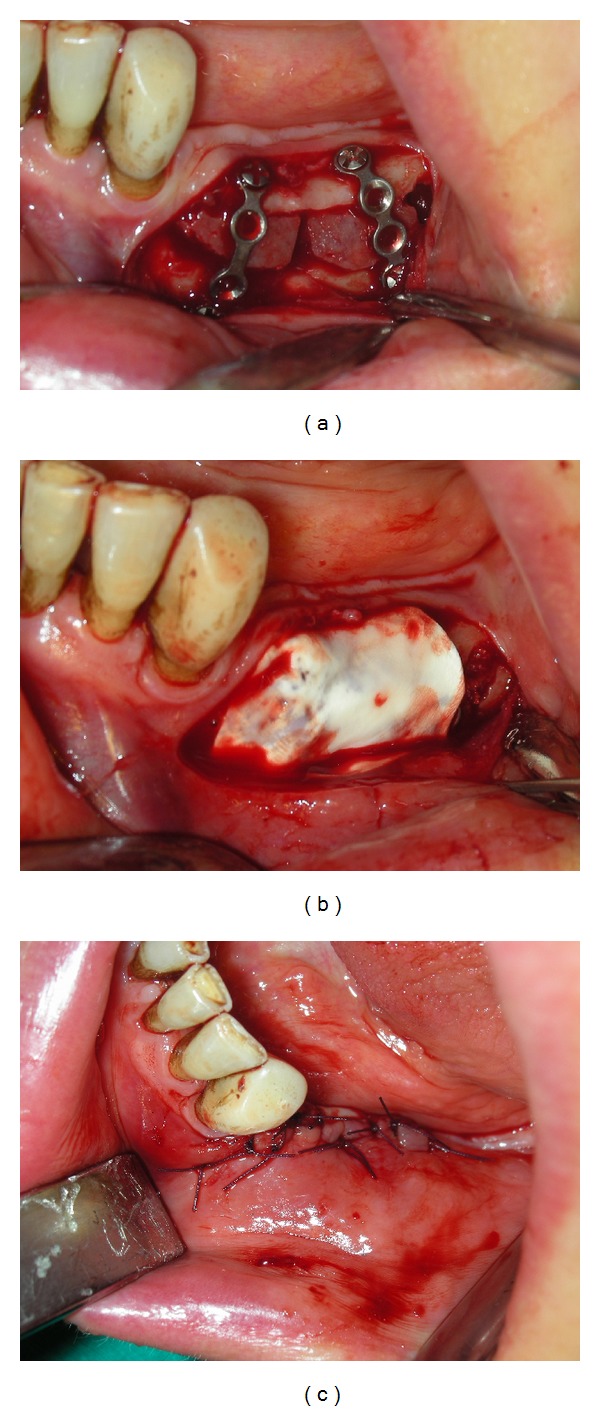
Sample of the grafted area covered with a resorbable barrier of pericardium (a, b, c).

Two weeks before bone augmentation and implant placement, all patients underwent oral hygiene instructions and professional debridement, when necessary. On the day of the augmentation procedure, the envelopes containing the randomized codes were opened. All patients received antibiotics prior to the surgery. Antimicrobial prophylaxis was obtained with the use of 1 gr of amoxicillin + clavulanic acid (Augmentin, GlaxoSmithKline, Brentford, Middlesex, UK) (or erythromycin 500 mg if allergic to penicillin), starting one day before surgery and for the following 4 days. All patients were treated under local anesthesia with intravenous sedation. A paracrestal incision was made through the buccal mucosa respecting the emergence of the mental nerve, and, as the full thickness flap was retracted, tension on the mental nerve was carefully avoided. The horizontal osteotomy was made at 4 mm from the mandibular canal using conventional surgical micromotor. Two oblique cuts were made in the coronal third of the mandibular bone with the mesial cut at least 2 mm distal to the last tooth in the arch Figures 1(a), 1(b), and 1(c). The osteotomies were completed with the use of bone chisels. The height of the osteotomized segment was at least 3 mm to allow the insertion of a stabilizing screw without risking the fracture of the distracted bone segment. The segment was elevated preserving the lingual periosteum, and according to the outcome of the randomization, the graft materials were modelled to the desired height and shape to fill the site and interposed between the raised fragment and the mandibular basal bone. Titanium miniplates and miniscrews (Gebruder Martin GmbH & Co., KG, Tuttlingen, Germany) were used to fix the osteotomized crestal bone to the basal bone. The grafted area was covered with a resorbable barrier of pericardium (Copios Pericardium Membrane Zimmer Dental, Switzerland) Figures 2(a), 2(b), and 2(c). Periosteal incisions were made to release the flaps coronally as needed and were sutured with Vicryl 5.0 sutures until the incisions were perfectly sealed. Patients were instructed to use Corsodyl gel 1% twice a day for 2 weeks and then 0.2 chlorhexidine mouthwashes twice a day for up to the second month, to avoid brushing and trauma on the surgical sites. Removable prostheses were not allowed. Patients were seen after 10 days for follow-up examinations and sutures removal. Patients were recalled for additional postoperative check-ups 1, 2, and 4 months after the augmentation procedure. Four months after augmentation, a CT scan was taken to plan implant placement.

### 2.2. Implant Placement

**Figure 3:**
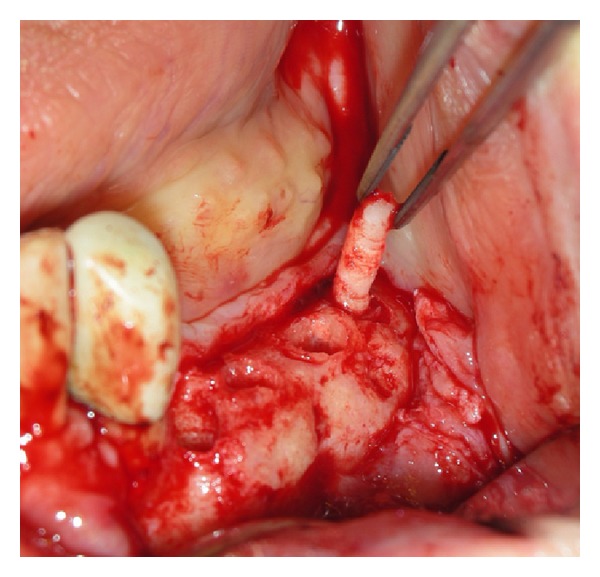
Sample of the bone core biopsies that were retrieved by using 2.9 mm diameter trephine bur.

Six months after the augmentation procedure, at the moment of dental implant surgery, miniplates were removed and the bone core biopsies were retrieved by using 2.9 mm diameter trephine bur (Komet 227b, Italy), and 72 implants (Spline Zimmer Dental, Switzerland) were inserted in situ, as shown in Figure 3. Drills with increasing diameters were used to prepare the implant sites. The surgical unit was settled with a torque of 25 Ncm. After the dental implant placement, the cover screws were placed and the flap closure was obtained with Vicryl 4.0. Patients were instructed to use 0.2% chlorhexidine mouthwash for 1 min twice a day for 2 weeks, to have a soft diet for 1 week, and to avoid brushing and trauma on the surgical sites. No removable prosthesis was allowed. Sutures were removed after 10 days.

### 2.3. Histological Procedure

Bone cores were retrieved, immediately stored in 10% buffered formalin, and processed to obtain thin ground sections using the Precise 1 Automated System (Assing, Rome, Italy). The specimens were dehydrated in a graded series of ethanol rinses and embedded in a glycol methacrylate resin (Technovit 7200 VLC, Kulzer, Wehrheim, Germany). After polymerization, the specimens were sectioned, along their longitudinal axis, with a high-precision diamond disc at about 150 *μ*m and ground down to about 30 *μ*m with a specially designed grinding machine. The slides were stained with acid fuchsin and toluidine blue and examined in normal transmitted light under a Leitz Laborlux microscope (Laborlux S, Leitz, Wetzlar, Germany). Histomorphometry of the percentages of newly formed bone, residual grafted material, and marrow spaces was carried out using a light microscope (Laborlux S, Leitz, Wetzlar, Germany) connected to a high resolution video camera (3CCD, JVC KY-F55B, JVC, Yokohama, Japan) and interfaced to a monitor and PC. This optical system was associated with a digitizing pad (Matrix Vision GmbH, Oppenweiler, Germany) and a histometry software package with image capturing capabilities (Image-Pro Plus 4.5, Media Cybernetics Inc., Immagini & Computer Snc, Milan, Italy). The same investigator made all the measurements.

### 2.4. Statistical Analysis

Data were evaluated by the Shapiro-Wilk test. All the data are presented as mean +/− standard deviations (SD); statistically significant differences were accepted as *P* < 0.05.

## 3. Results

The failures and complications that occurred during the entire study period were limited. In one patient treated with the Puros bone block, exposure of a titanium plate 2 months after surgery occurred; it was treated by removing the plaque, and then a satisfactory healing was achieved. In two patients treated with autologous bone from mental symphysis, a temporary paresthesia of the anterior region of the mandible was appreciated and treated by drug solution, Dobetin 5000 mcg 1 time per day for 1 week and 3 doses in the second week.

### 3.1. Histological Results

#### 3.1.1. Control Group

**Figure 4:**
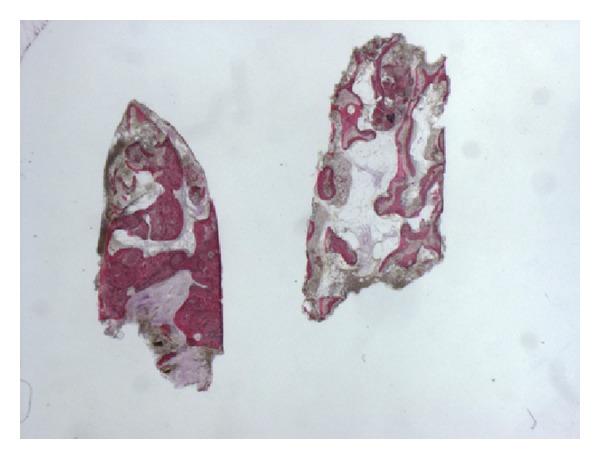
Grafted bone, almost completely surrounded by newly formed bone can be observed. Acid fuchsin-toluidine blue; original magnification 60x.

**Figure 5:**
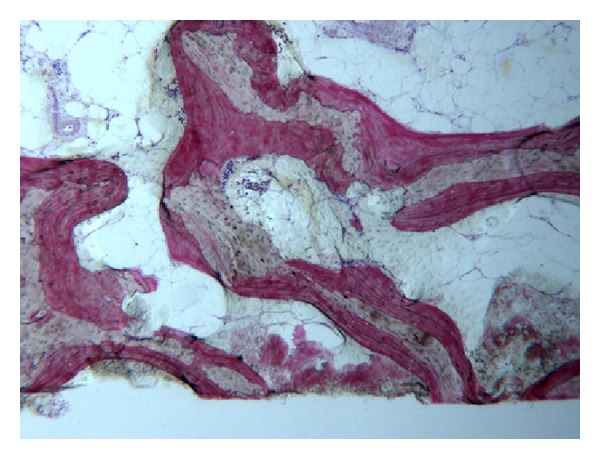
The autogenous bone block presents marked staining differences from the host trabecular bone and specifically, it shows a lower affinity for the stains. The block is surrounded by newly formed bone. Acid fuchsin-toluidine blue; original magnification 40x.

**Figure 6:**
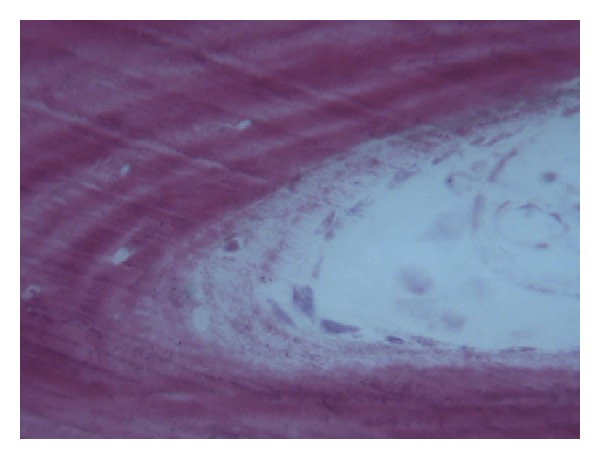
A rim of osteoblasts depositing osteoid matrix is evident. Acid fuchsin-toluidine blue; original magnification 200x.

**Figure 7:**
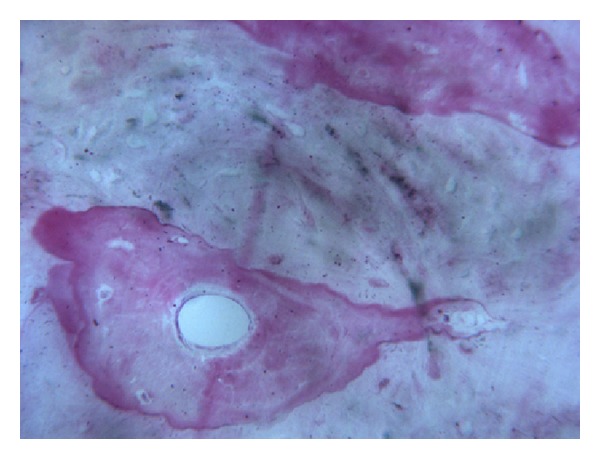
An osteon in the vicinity of grafted bone can be seen. Acid fuchsin-toluidine blue; original magnification 200x.

In the control group, a significant amount of grafted bone, almost completely surrounded by newly formed bone, was observed (Figure 4). The autologous grafted bone showed irregularly shaped margins, probably due to the remodeling process. The demarcation line (cementing line) between grafted bone and newly formed bone was evident (Figure 5). In some areas, bone remodeling was conceivable with a rim of osteoblasts depositing osteoid matrix (Figure 6). Osteons in the vicinity of grafted bone could be observed (Figure 7). No signs of inflammatory infiltrate were present.

#### 3.1.2. Test Group

**Figure 8:**
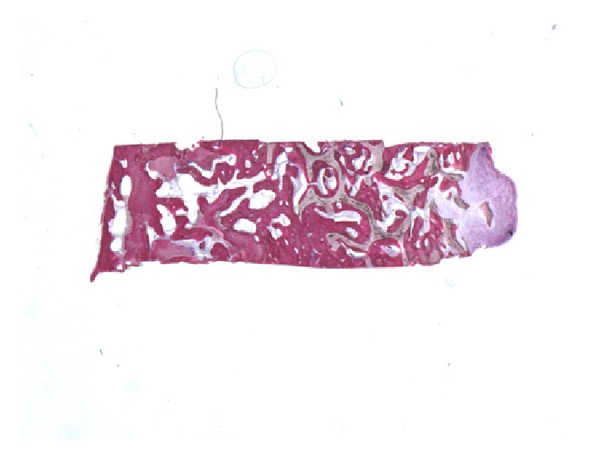
A good amount of newly formed bone can be observed. Acid fuchsin-toluidine blue; original magnification 6x.

**Figure 9:**
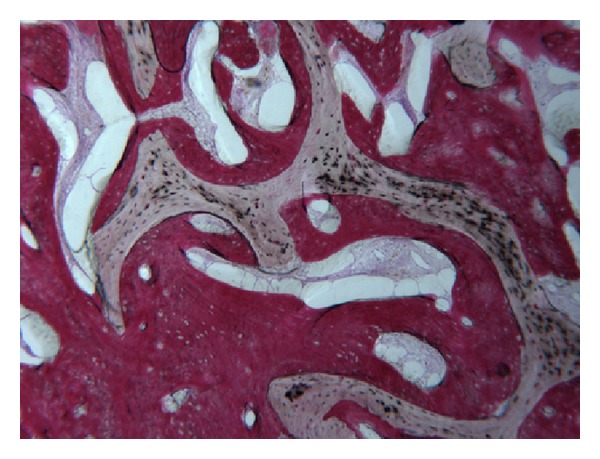
The bovine bone block is surrounded by newly formed bone. A tight contact between the grafted material and the regenerated bone without any interposition of fibrous tissue can be observed. Acid fuchsin-toluidine blue; original magnification 40x.

**Figure 10:**
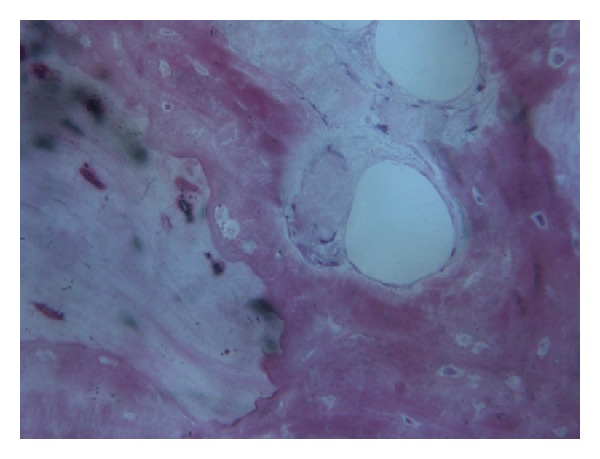
Large osteocyte lacunae in contact with the grafted material are present. Acid fuchsin-toluidine blue; original magnification 200x.

**Figure 11:**
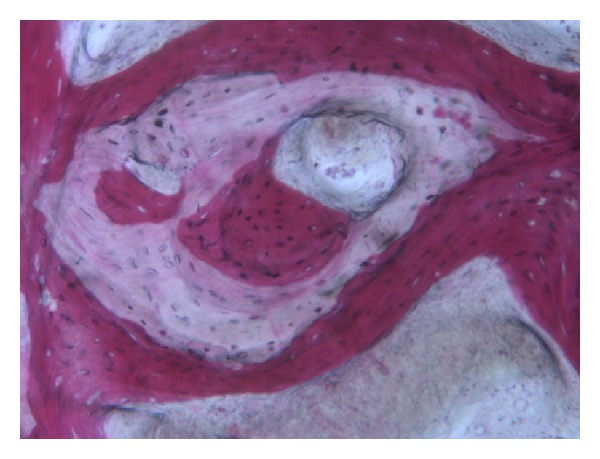
New bone can be seen in the inner and outer portions of a residual grafted particle. Acid fuchsin-toluidine blue; original magnification 100x.

In all the analyzed samples, a good amount of newly formed bone could be observed (Figure 8). A tight contact between the grafted material and the regenerated bone, without any interposition of fibrous tissue, was found (Figure 9). The newly formed bone had a high affinity for dyes and was acid fuchsine positive; therefore, a highly stained line was observed at the grafting material-new bone interface. In many fields, it was possible to observe the presence of large osteocytes lacunae in contact with the grafted material (Figure 10). Some trabeculae of grafted material were bridged by newly formed bone, which was observed both in the inner and outer portions of some biomaterial particles (Figure 11). Marrow stromal cells and blood vessels were found inside the marrow spaces. In some fields, there was a modest amount of inflammatory infiltrate. No osteoclasts were observed around the graft particles.

The histomorphometric results are summarized in Tables 1, 2, and 3.

There was no statistically significant difference between the two groups in terms of amount of new bone, 31.47 ± 2.2 versus 30.6 ± 3.7% (*P* = 0.5362 has been recorded, while there was a statistically significant difference in the percentage of residual grafted material higher in test group (Table 2).

## 4. Discussion

This study is designed to evaluate how a bone substitute material may offer some advantages in the place of autogenous bone grafts harvested from the mental symphysis in the treatment of atrophic posterior mandibles. Moreover, the authors proposed a novel technique for localized vertical bone augmentation using an interpositional bone block representing a valuable and predictable surgical alternative technique for posterior mandible atrophic ridge.

In 2006, Jensen retrospectively evaluated the crestal stability of alveolar augmentation using an interpositional bone graft for dental implant restorations and found a good stability after 4-year follow-up. In 2008, Felice et al. produced a series of clinical investigations for analyzing the effectiveness of this technique in relation to the use of biomaterial, and also in this case the results appeared satisfactory [12–15].

Another main aspect is the choice of the graft material to be used. In 2009, Felice et al. performed a randomized controlled clinical trial to evaluate two different kinds of graft materials: bone form iliac crest and bovine anorganic bone. There were ten selected partially edentulous patients having 5–7 mm of residual crystal height above the mandibular canal. Four months after bone grafting, a bone core was retrieved from each side using a 3 mm external diameter trephine for the histological evaluation. The histomorphometric data showed the only statistically significant difference in the mean percentage of residual graft (between 10% and 13%, *P* values between 0.008 and 0.009) that was greater in the Bio-Oss group, while there were no statistically significant differences in the percentage of newly formed bone and in the marrow space between the two groups. Also the clinical outcomes present in the literature are very interesting [6, 12, 14–18]. In 2006, Jensen published a retrospective study to evaluate the crystal stability of alveolar augmentation using an interpositional bone graft for dental implant restorations. Eight patients with 10 graft sites were followed from 1 to 4 years with panographic evaluation to determine if dimension changes of the alveolar graft sites had occurred. The author described a little loss of crystal height and 20 of the 22 implants confirmed high stability at the follow up evaluation. These results were later confirmed by various studies conducted by the group of Felice et al. (2008, 2009) and the results of this study confirmed the possibility of considered this surgical technique like predictable one [2, 4, 7, 12, 16, 18].

The sandwich osteotomy can be considered an alternative to other bone augmentation techniques presented in the recent literature. Although one study determined that interpositional bone grafting and alveolar distraction yielded statistically similar results in regard to buccolingual width, it later stated that bone grafting may form wider bone. No mention was made of the advantage gained with the copious blood supply by the sandwich osteotomy technique [14–19].

Other studies have shown that fewer cases of dehiscence were observed with the sandwich osteotomy than with techniques using only graft or titanium mesh. Laviv et al. recently reported that in 10 patients treated with a similar technique, vertical gain ranged from 3 to 6 mm over a 4-year period [16]. Authors stated that efforts to displace the segment greater than 5 mm “may not only risk the potential for vascular embarrassment by detaching periosteal blood supply, but also can excessively rotate the segment palatally, compromising aesthetic gingival projection.” In the anterior maxilla, it has been shown that one of the disadvantages could be the reduced extensibility of the palatal mucoperiosteum that does not allow a vertical increase of more than 10 mm to be done [6, 18–20]. In a letter to the editor, Robiony et al. suggest that the vertical movement could be extended more than the 10 mm proposed by Jensen, but only in the canine and premolar zones. They described their experience with 25 patients and demonstrated that the technique can be successful without compromising vascular supply and esthetics [20–24]. All those studies clearly demonstrated, even with some limitations, how the use of this technique, by expert surgeons, might be safe and predictable giving less discomfort for the patients [14, 22, 25].

In the present study, in order to offer our patients a less invasive surgery, a biomaterial has been compared to autologous bone and the difference in newly formed bone percentages was not statistically significant. During the histomorphometric evaluation, the percentage of newly formed bone was found to be lower in the test group (28.9/19.5); this meant a slower integration of the grafted material, which is not clinically appreciable; therefore, the use of autologous bone blocks does not seem to provide particular advantages.

## 5. Conclusion

The results of the present investigation encourage further studies on the present topic. However, other clinical trials are needed, with a greater number of patients. The results reported in the literature and this preliminary study underline how the interposition technique seems to be a valid therapeutic option in the treatment of vertical atrophy of the posterior mandible. Both graft materials gave good results in relation to this type of surgical technique; the use of Puros bone block allograft represents a less invasive alternative for the patients. In the future, it would be interesting to compare this technique with short implants and to record those results over the long term.

## Figures and Tables

**Table 1:** 

Group	Obs.	Mean	Std. error	Std. deviation	95% conf. I.
Group Ctrl	12	31.47	7155495	2.262.766	29.85131 33.08868

Group test	12	30.6	117.936	3.729.462	27.9321 33.2679

Newly formed bone, *t* value = 0.6307, and *P* value = 0.5362. There is no statistically significant difference.

**Table 2:** 

Group	Obs.	Mean	Std. error	Std. deviation	95% conf. I.
Group Ctrl	12	19.56	1.320.959	417.724	16.57178 22.54822

Group test	12	28.9	1.600.069	5.059.864	25.28039 32.51961

Residual graft material, *t* value = −4.5015, and *P* value = 0.0003. There is statistically significant difference.

**Table 3:** 

Group	Obs	Mean	Std. error	Std. deviation	95% conf. I.
Group Ctrl	12	48.97	1.878.241	5.939.519	44.72112 53.21888

Grouptest	12	41.28	1.888.491	5.971.934	41.84335 48.40665

Marrow space, *t* value = 2.8872, and *P* value = 0.0098. There is statistically significant difference.
